# Comparing the Risk of Epilepsy in Patients With Simple Congenital Heart Diseases: A Prospective Cohort Study

**DOI:** 10.1111/cns.70230

**Published:** 2025-02-07

**Authors:** Lei Chen, Zuyao Yang, Shuming Ji, Tingting Song, Hua Li, Yusha Tang, Yucheng Chen, Yajiao Li

**Affiliations:** ^1^ Department of Neurology, Joint Research Institution of Altitude Health, West China Hospital Sichuan University Chengdu China; ^2^ Sichuan Provincial Engineering Research Center of Brain‐Machine Interface Chengdu China; ^3^ Sichuan Provincial Engineering Research Center of Neuromodulation Chengdu China; ^4^ JC School of Public Health and Primary Care The Chinese University of Hong Kong Hong Kong China; ^5^ Department of Clinical Research Management, West China Hospital Sichuan University Chengdu China; ^6^ Department of Cardiology, West China Hospital Sichuan University Chengdu China

**Keywords:** epilepsy, incidence rate, patent foramen ovale, simple congenital heart diseases

## Abstract

**Aims:**

Simple congenital heart diseases (CHD) are associated with various central nervous system diseases, including epilepsy. This study aimed to compare the risk of epilepsy in patients with different types of simple CHD.

**Methods:**

In this prospective cohort study, from January 2008 to June 2022, patients with atrial septal defect (ASD), patent foramen ovale (PFO), ventricular septal defect (VSD), and patent ductus arteriosus (PDA) were recruited at the Registration Center of CHD in West China Hospital. Follow‐up was conducted yearly until the diagnosis of epilepsy, loss to follow‐up, or end of study. The outcomes included a comparison of epilepsy incidence according to different simple CHD types and a risk assessment of developing epilepsy. Multivariable Poisson regression was performed to adjusted factors of demographics and disease history.

**Results:**

Of 10,914 patients who met the inclusion criteria, 108 were diagnosed with epilepsy at an average follow‐up of 2.19 years. Epilepsy incidence in patients with PFO, VSD, PDA, and ASD was 8.58/1000, 4.85/1000, 3.98/1000, and 2.63/1000 person‐years, respectively. Compared with ASD patients (reference group), the risk ratios (95% confidence intervals) in patients with PFO, VSD, and PDA were 3.28 (2.00–5.43), 1.47 (0.79–2.68), and 1.46 (0.70–2.82), respectively. Subgroup analyses determined that patients with simple CHD who underwent CHD surgery demonstrated a lower risk of epilepsy than those who did not.

**Conclusion:**

Among the major types of simple CHD, PFO was associated with a significantly higher risk of epilepsy, while the risk was reduced in those who underwent PFO closure procedures.

## Graphical abstract

Different types of simple congenital heart diseases showed significantly different risks of epilepsy. Patients with patent foramen ovale had a significantly higher risk of epilepsy than those with other simple congenital heart disease types. Patients who underwent surgery for congenital heart disease had a lower risk of epilepsy than those who did not. PFO closure may have a significant effect on reducing the risk of epilepsy.

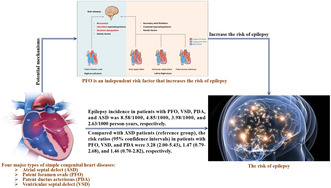

**Abbreviations**
ASD: atrial septal defect
CHD: congenital heart diseases
CI: confidence interval
CMR: cardiac magnetic resonance
CoA: coarctation of the aorta
CSD: cortical spreading inhibition
CT: computed tomography
CTTE: contrast transthoracic echocardiography
EEG: electroencephalograph
FLAIR: fluid‐attenuated inversion recovery
ILAE: International League Against Epilepsy
IR: incidence rate
IRD: incidence rate difference
LV: left ventricle
LVEF: LV ejection fraction
MRI: magnetic resonance imaging
OSA: obstructive sleep apnea
PAP: pulmonary artery pressure
PDA: patent ductus arteriosus
PET: positron emission tomography
PFO: patent foramen ovale
PNES: psychogenic non‐epileptic seizures
PWE: patients with epilepsy
RR: risk ratios
RV: right ventricle
RVOTO: RV outflow tract obstruction
SPECT: single‐photon emission CT
UVH: univentricular heart
V‐EEG: video‐EEG
VSD: ventricular septal defect

## 1 Introduction

Congenital heart diseases (CHD) are the most common congenital disorders among newborns [1]. In contrast to serious and moderately complex CHD, which presents with severe clinical symptoms and high mortality [2], simple CHD is associated with mild symptoms and has a survival rate equivalent to that of the general population [3]. However, various studies have shown that simple CHD may result in damage to the central nervous system in the long term and significantly increase the risk of neurological diseases [4, 5], such as neurodevelopmental disorders [6, 7], migraine [8, 9, 10], cryptogenic stroke [11, 12], and epilepsy [13].

Specifically, epilepsy is a common chronic neurological disorder affecting over 70 million people worldwide [14]. Nearly 40% of patients with epilepsy (PWE) have an unclear etiology, which increases the difficulty of curing epilepsy and results in a heavy disease burden for PWE [15, 16]. Therefore, exploring the possible etiology is an important area of epilepsy research that may assist to provide more specific etiological screening and effective treatment for PWE. Some studies have found that simple CHD can increase the risk of epilepsy in the long term. For instance, a cohort study from Denmark found that the risk of epilepsy among patients with mild and moderate CHD was 2.1–3.8 times that in the matched general population (the mild and moderate CHD categories in this study were ASD, VSD, PDA, and pulmonary stenosis) [13]. A nationwide cohort study from Sweden found that the risk of epilepsy among patients with CHD was 3.6 times that among matched controls [17]. In a separate study, we also found that the prevalence of PFO in PWE was 1.6 times higher than that in matched controls [18].

According to epidemiological findings, atrial septal defect (ASD), patent foramen ovale (PFO), ventricular septal defect (VSD), and patent ductus arteriosus (PDA) are the dominant simple congenital heart defects with abnormal cardiac shunt [19, 20]. Some studies on pathogenic mechanisms have suggested that simple CHD associated with neurological diseases might result from paradoxical embolism and cerebral hypoxia caused by abnormal cardiac shunt [21, 22, 23, 24]. However, in all patients with simple CHD and abnormal cardiac shunt, which may be a pathological mechanism that increases the risk of epilepsy, an observational comparison of the incidence of epilepsy over time is currently unavailable. Therefore, we conducted a prospective cohort study to compare the risk of epilepsy in patients with different types of simple CHD.

## 2 Methods

**FIGURE 1:**
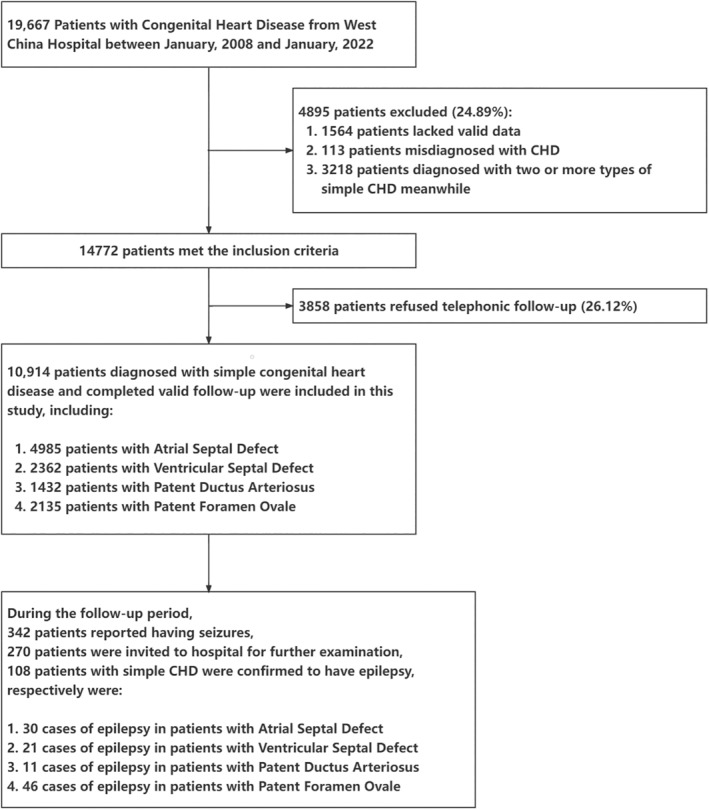
Implementation and follow‐up procedure of simple congenital heart diseases cohort.

Since January 2008, a continuous registration database established in the West China Hospital of Sichuan University included patients with simple CHD, mainly including four types of simple CHD: ASD, VSD, PDA, and PFO [25]. In this prospective cohort study, patients diagnosed with simple CHD were recruited through a standardized diagnostic procedure and required to complete a physical examination and symptom assessment at baseline. Our study excluded patients who were diagnosed with two or more types of simple CHD meanwhile, those who reported a history of seizures or epilepsy at the time of registration, those without valid data or contact information during the database checkup, and those with a misdiagnosis of simple CHD. A flowchart depicting the process of recruitment and inclusion and exclusion criteria is shown in Figure 1.

### 2.1 Diagnostic Procedures

The diagnostic criteria of simple CHD were based on the European Society of Cardiology (ESC) guidelines on the management of grown‐up CHD (ESC, 2010 version) [26], and the American Society of Echocardiography and Society for Cardiac Angiography and Intervention (ASE and SCAI, 2015 version) [27]; in addition, epilepsy was confirmed using the standards of the International League Against Epilepsy (ILAE) [28], and the identification of drug‐resistant epilepsy was confirmed according to the ILAE definition of drug‐resistant epilepsy (2010 version) [29]. Detailed diagnostic procedures of simple CHD and epilepsy are shown in the first section of the Supporting Information S1, including computed tomography (CT), cardiac magnetic resonance (CMR) imaging, transthoracic echocardiography (TTE), electrocardiogram (ECG), electroencephalography (EEG), and magnetic resonance imaging (MRI), etc.

### 2.2 Clinical Information Collection

All patients recruited in our study reported their clinical information at baseline, including sex, age, ethnicity, smoking status, alcohol consumption, and chronic comorbidity (e.g., hypertension, diabetes, stroke, and migraine). Disease history during infancy was recorded by asking parents or relatives about hypoxia at birth, premature birth, history of febrile convulsion, and congenital anomalies. Additionally, whether the patients underwent the corresponding CHD surgery before recruitment or during the follow‐up period was recorded in the database. Information about patients who were too young to communicate or had cognitive impairment was provided by their guardians.

### 2.3 Follow‐Up Process and Outcome

From January 2008, a follow‐up telephonic survey was conducted at least once a year from the diagnosis of simple CHD until the diagnosis of epilepsy, loss to follow‐up, or June 31, 2022 (end of the study), whichever occurred first. Patients who refused telephonic follow‐up were excluded.

The primary outcome of this study was the diagnosis of epilepsy. During each telephonic survey during the whole follow‐up period, patients were asked whether they had experienced seizures in the past year, and those who provided a “yes” response were invited to the hospital for further examination. During this process, patients were asked to provide as much detail as possible regarding any symptoms they believed may be indicative of epileptic seizures. They were also encouraged to review the specific scenes and frequency of such symptoms over the past year. In cases where other family members or witnesses were present during a seizure, a telephone review was conducted. These measures were used to avoid possible missed and delayed diagnoses of epilepsy.

The diagnosis of epilepsy was confirmed or excluded by neurologists following the 2005 version of ILAE's guidelines (or after 2014, the 2014 version of guidelines) [28, 30]. This was conducted by a team of neurologists who recorded a comprehensive disease history of the patient, a series of clinical examinations, and a detailed review of the seizure symptoms to achieve an accurate diagnosis. Besides, chest tightness, psychogenic non‐epileptic seizures (PNES), cardiogenic syncope, and other diseases that can mimic epileptic seizures were also excluded from this process.

For patients diagnosed with epilepsy, seizure characteristics, including age at onset, status epilepticus, etiology, seizure classification, seizure duration, seizure frequency, and use of anti‐seizure medications, were recorded. Based on these seizure characteristics and the corresponding diagnostic criteria, our neurologist team determined whether the patients had epileptic loss of consciousness, rolandic epilepsy, or refractory epilepsy.

### 2.4 Statistical Analyses

The multiple imputation method was used to complete the missing values of the seizure characteristics for CHD patients with epilepsy during the long‐term follow‐up. Mean ± standard deviation (SD) was used to describe the continuous data, and frequency (percentage) was used to describe the counting data. All CHD patients were divided into four groups and described independently; all continuous data were tested for normality and compared between groups using the appropriate test method (Analysis of variance (ANOVA) was suitable for continuous variables with normal distribution; Kruskal‐Wallis (K‐W) test was used for continuous variables that did not follow the normal distribution or ordered categorical data; and Chi‐square test or Fisher's exact test was used for categorical data).

Patient characteristics were described according to the different categories of simple CHD. Age was stratified into three groups: ≤ 18, 18–60, and > 60 years. Seizure frequency was classified as follows: < 1, 1–12, and > 12 seizures per year. Seizure duration was classified as follows: < 1, 1–3, and > 3 min. Based on seizure presentation, epilepsy was classified into focal, general, and unknown types. The manifestations of seizures in PWE were also compared across different categories of simple CHD. We calculated and used the incidence rate (IR), expressed as the number of cases per 1000 person‐years, to quantify the incidence of epilepsy. Differences in IR among patients with different types of simple CHD were also determined. Poisson regression was used to estimate the risk ratio (RR) and 95% confidence interval (CI). Clinical characteristics were considered as confounding factors in the regression model.

To explore whether CHD surgery modified the risk of epilepsy, subgroup analysis was conducted regarding whether patients with different types of simple CHD underwent the corresponding surgery. In theory, because simple CHD is concomitant at birth, the exposure time should be equal to the actual age. The main analysis considered potential heterogeneity in age distribution between different simple CHD types and hysteresis in long‐term effects on the risk of epilepsy. Therefore, we used the follow‐up duration after diagnosis, instead of the actual age, as the exposure duration. However, in the sensitivity analysis, the actual age of patients was used as a substitute for the follow‐up duration. Statistical analyses were performed using R. version 4.2.1 (R Foundation for Statistical Computing, using packages such as mice, fmsb, and tidyverse). All *p* values were two‐tailed, and statistical significance was set at 0.05.

### 2.5 Ethics and Data Availability

This study was approved by the Ethics Committee of the West China Hospital of Sichuan University and reported in accordance with the Strengthening the Reporting of Observational Studies in Epidemiology (STROBE) Statement, as shown in Supporting Information S1. All the participants provided written informed consent. As the participants did not provide explicit consent for the public sharing of their data, the original individual data are unavailable to people other than the investigators of this study.

## 3 Results

### 3.1 Characteristics of Patients With Simple CHD

Between 2008 and 2022, 10,914 patients diagnosed with simple CHD were eligible for inclusion criteria and enrolled. The average follow‐up period was 2.19 years (min–max: 3 days–13.25 years), with an overall 23.86 thousand person‐years of follow‐up (Table S1). During this study period, 108 patients were diagnosed with epilepsy: 30 (0.60%), 11 (0.77%), 21 (0.89%), and 46 (2.15%) with ASD, PDA, VSD, and PFO, respectively. The characteristics of patients with simple CHD are presented in Table 1. The proportion of female patients was approximately 50% in both VSD and PFO patients but increased to more than 65% in patients with ASD and PDA. The average age of the patients increased in the following order: VSD, PDA, ASD, and PFO, with the results of normality test and the distribution between groups shown in Figures S1 and S3. Patients with PFO had a higher proportion of hypertension, diabetes, and stroke compared to those with other types, although the surgical acceptance rate in the PFO group was lower. In addition, the prevalence of birth hypoxia and febrile convulsion in different types of simple CHD was similar, but the prevalence of congenital anomalies in patients with PFO was significantly higher than that in patients with other types of simple CHD.

**TABLE 1:** The characteristics of patients with simple congenital heart diseases.

	Total no. of cases	Classification of simple congenital heart diseases^a^				*p* ^b^
		ASD	PDA	VSD	PFO	
*Number of patients*	10,914 (100)	4985 (45.68)	1432 (13.12)	2362 (21.64)	2135 (19.56)	
*Demographic characteristics*						
Gender						
Male	4292 (39.33)	1622 (32.54)	358 (25.00)	1275 (53.98)	1037 (48.57)	< 0.001
Female	6622 (60.67)	3363 (67.46)	1074 (75.00)	1087 (46.02)	1098 (51.43)	
Age, median (IQR), years	35 (14–54)	40 (25–54)	30 (13–47)	14 (8–32)	54 (29–69)	
≤ 18	3118 (28.57)	867 (17.39)	481 (33.59)	1356 (57.41)	414 (19.39)	< 0.001
18–60	5986 (54.85)	3355 (67.30)	830 (57.96)	932 (39.46)	869 (40.70)	
> 60	1810 (16.58)	763 (15.31)	121 (8.45)	74 (3.13)	852 (39.91)	
Minority, ref. = Han	1047 (9.59)	507 (10.17)	224 (15.64)	97 (4.11)	219 (10.26)	< 0.001
*Behavioral factors*						
Smoking	850 (7.79)	192 (3.85)	75 (5.24)	216 (9.145)	367 (17.19)	< 0.001
Alcohol	510 (4.67)	214 (4.29)	80 (5.59)	58 (2.456)	158 (7.40)	< 0.001
*Complications*						
Hypertension	1411 (12.93)	511 (10.25)	122 (8.52)	93 (3.94)	685 (32.08)	< 0.001
Diabetes	564 (5.17)	213 (4.27)	39 (2.72)	25 (1.06)	287 (13.44)	< 0.001
Stroke	510 (4.67)	110 (2.21)	19 (1.33)	27 (1.14)	354 (16.58)	< 0.001
Migraine	139 (1.27)	92 (1.85)	15 (1.05)	5 (0.21)	27 (1.27)	< 0.001
CHD Surgery	7277 (66.68)	3959 (79.42)	1145 (79.96)	1892 (80.10)	281 (13.16)	< 0.001
*Diseases history at birth or during infancy* ^c^						
Birth hypoxia	163 (1.49)	67 (1.34)	28 (1.96)	33 (1.40)	35 (1.64)	0.349
Birth preterm	212 (1.94)	83 (1.67)	35 (2.44)	40 (1.69)	54 (2.53)	0.036
Febrile convulsion	144 (1.32)	58 (1.16)	23 (1.61)	39 (1.65)	24 (1.12)	0.216
Congenital anomaly^d^	520 (4.77)	201 (4.03)	59 (4.12)	103 (4.36)	157 (7.35)	< 0.001
*Incident epilepsy*	108 (0.99)	30 (0.60)	11 (0.77)	21 (0.89)	46 (2.15)	< 0.001

Abbreviations: ASD, atrial septal defect; IQR, interquartile range; PDA, patent ductus arteriosus; PFO, patent foramen ovale; VSD, ventricular septal defect.

^a^ Data are expressed as no. (%) unless otherwise indicated.

^b^ Chi‐square test and Fisher's exact test were used to compare the difference in frequency distribution of four simple congenital heart diseases, and Kruskal‐Wallis test was used to compare the difference in age distribution of the above.

^c^ Recorded subject's diseases history under 6 years old by themselves or family member through memory.

^d^ An abnormality of structure, function, or metabolism present at birth that results in physical or mental disability, or is fatal, except congenital heart diseases.

### 3.2 Comparison of the Risk of Epilepsy

The incidence of epilepsy was 8.58, 2.63, 3.98, and 4.85/1000 person‐years in patients with PFO, ASD, PDA, and VSD, respectively. Compared to patients with ASD (reference group with the lowest epilepsy incidence), those with PFO had the largest difference in IR with statistical significance (IR difference [IRD] = 5.95, 95% CI: 3.30–9.60). Non‐significant differences occurred in IR with PDA (IRD = 1.34, 95% CI: −1.19–3.88) and VSD (IRD = 2.22, 95% CI: −0.06–4.50). Poisson regression adjusted for multiple confounding factors showed that, compared to ASD, PFO was associated with an increased risk of epilepsy (RR = 3.28, 95% CI: 2.00–5.43). However, the increased risk with PDA (RR = 1.46, 95% CI: 0.70–2.82) and VSD (RR = 1.47, 95% CI: 0.79–2.68) was not statistically significant. The adjusted incidence rate based on the Poisson regression was 2.63, 5.81, 7.13, and 28.14/1000 person‐years in patients with ASD, PDA, VSD, and PFO, respectively. The details are shown in Table 2.

**TABLE 2:** The risk ratio, incidence rate, and incidence rate difference of epilepsy in patients with simple congenital heart diseases.

	Person‐years	No. of CHD cases	No. of epilepsy	Adjusted risk ratio (95% CI)^a^	Incidence rate/1000 person‐years	Incidence rate difference/1000 person‐years	Adjusted incidence rate/1000 person‐years
Classification of simple CHD							
ASD	11400.29	4985	30	1	2.63	0	2.63
PDA	2766.59	1432	11	1.46 (0.70 to 2.82)	3.98	1.34 (−1.19 to 3.88)	5.81
VSD	4330.85	2362	21	1.47 (0.79 to 2.68)	4.85	2.22 (−0.06 to 4.50)	7.13
PFO	5362.25	2135	46	3.28 (2.00 to 5.43)	8.58	5.95 (3.30 to 8.60)	28.14
Simple CHD with a surgery history							
ASD	9004.82	3959	21	1	2.33	0	2.33
PDA	2176.21	1145	8	1.50 (0.62 to 3.29)	3.68	1.34 (−1.39 to 4.08)	5.52
VSD	3518.96	1892	15	1.48 (0.68 to 3.17)	4.26	1.93 (−0.45 to 4.31)	6.30
PFO	824.17	281	5	1.92 (0.58 to 5.26)	6.07	3.73 (−1.68 to 9.14)	11.65
Simple CHD without a surgery history							
ASD	2395.47	1026	9	1	3.76	0	3.76
PDA	590.37	287	3	1.25 (0.28 to 4.21)	5.08	1.32 (−4.93 to 7.58)	6.35
VSD	811.88	470	6	1.41 (0.46 to 4.01)	7.39	3.63 (−2.77 to 10.04)	10.42
PFO	4538.08	1854	41	2.55 (1.27 to 5.68)	9.03	5.28 (1.58 to 8.98)	23.03

Abbreviations: ASD, atrial septal defect; CI, confidence interval; PDA, patent ductus arteriosus; PFO, patent foramen ovale; VSD, ventricular septal defect.

^a^ The risk ratio was adjusted for gender, age, minority, smoking, alcohol, hypertension, diabetes, stroke, migraine, birth hypoxia, birth preterm, febrile convulsion, congenital anomaly.

In the subgroup analyses, patients with simple CHD who underwent the corresponding surgery presented a lower incidence of epilepsy than those who did not. Regardless of the type of simple CHD, patients who underwent surgery seemed to have a lower risk of epilepsy than those who did not, which was more evident in patients with PFO. For patients with PFO, the RR value was not significant in the underwent CHD surgery subgroup (RR = 1.92, 95% CI: 0.58–5.26) but remained significant in the no CHD surgery subgroup (RR = 2.55, 95% CI: 1.27–5.68).

Associations between other factors and the incidence of epilepsy are presented in Table 3. Among them, increasing age and accompanying with diabetes were protective factors for the risk of epilepsy, while ethnic minority patients, patients accompanied with stroke, and patients with hypoxia at birth were risk factors for epilepsy. There were no significant differences in epileptic seizure features among patients with different types of simple CHD, except in age at onset of epilepsy and seizure classification. Among them, the difference in onset age was mainly reflected as that the VSD patients had the youngest onset age (median onset age with IQR: 12, 5–33), while the PFO patients had the oldest onset age (median onset age with IQR: 30, 15–59). And the main difference in seizure classification was that patients with PFO, ASD, and PDA had a relatively high proportion of generalized seizures, while VSD patients had a relatively high proportion of focal seizures, as shown in Table 4. The normality test and distribution of age at onset of epilepsy in patients with various types of simple CHD are shown in Figures S2 and S4.

**TABLE 3:** The association of demographic characteristics, behavioral variables, complications, and disease history with the epilepsy incidence.

	No. of CHD cases	No. of epilepsy cases	Independent risk ratio
*Patent foramen ovale*	2135	46	3.15 (1.99–4.91)
*Demographic characteristics*			
Gender, ref. = male	6622	58	0.85 (0.55–1.32)
Age (years)			
≤ 18	3118	25	1
18–60	3056	43	0.77 (0.46–1.33)
> 60	1810	16	0.28 (0.13–0.59)
Minority, ref. = Han	1047	16	2.53 (1.42–4.22)
*Behavioral factors*			
Smoking	850	16	1.60 (0.83–2.96)
Alcohol	510	3	0.50 (0.12–1.40)
*Complications*			
Hypertension	1411	21	0.78 (0.44–1.34)
Diabetes	564	5	0.35 (0.12–0.82)
Stroke	510	21	4.14 (2.34–7.09)
Migraine	139	2	0.83 (0.14–2.69)
*Diseases history at birth or during infancy* ^a^			
Birth hypoxia	163	6	3.91 (1.46–8.67)
Birth preterm	212	3	0.95 (0.22–2.77)
Febrile convulsion	144	4	2.05 (0.62–5.04)
Congenital anomaly^b^	520	8	1.45 (0.62–2.96)

^a^ Recorded subject's diseases history under 6 years old by themselves or family member through memory.

^b^ An abnormality of structure, function, or metabolism present at birth that results in physical or mental disability, or is fatal, except congenital heart diseases.

**TABLE 4:** The characteristics of seizure in patients with epilepsy and simple congenital heart diseases.

	Classification of simple congenital heart diseases^a^				*p* ^b^
	ASD (*N* = 30, 27.78%)	PDA (*N* = 11, 10.19%)	VSD (*N* = 21, 19.44%)	PFO (*N* = 46, 42.59%)	
Onset age, median (IQR), years	26 (16–36)	23 (11–42)	12 (5–33)	30 (15–59)	0.025
Status epilepticus	7 (23.33)	2 (18.18)	3 (14.29)	18 (39.13)	0.152
Refractory epilepsy	4 (13.33)	2 (18.18)	2 (9.52)	3 (6.52)	0.512
Seizure absence, ref. = Unknown or without	25 (83.33)	10 (90.91)	16 (76.19)	37 (80.44)	0.808
Seizure movement, ref. = Unknown or without	18 (60.00)	9 (81.82)	12 (57.14)	36 (78.26)	0.159
Etiology unknown, ref. = Definite	21 (70.00)	8 (72.73)	16 (76.19)	29 (63.04)	0.784
Seizure classification					
Unknown	3 (10.00)	3 (27.27)	7 (33.33)	4 (8.70)	0.043
Focal	13 (43.33)	3 (27.27)	9 (42.86)	17 (36.96)	
General	14 (46.67)	5 (45.46)	5 (23.81)	25 (54.35)	
Seizure duration after taken ASMs^c^					
< min	19 (63.33)	7 (63.64)	8 (38.10)	26 (56.52)	0.089
1–3 min	6 (20.00)	3 (27.27)	6 (28.57)	16 (34.78)	
> 3 min	5 (16.67)	1 (9.09)	7 (33.33)	4 (8.70)	
Seizure frequency after receiving ASMs^c^					
< 1 per year	11 (36.67)	4 (36.36)	12 (57.14)	28 (60.87)	0.170
1–12 per year	12 (40.00)	4 (36.36)	6 (28.57)	14 (30.44)	
> 1 per month	7 (23.33)	3 (27.27)	3 (14.29)	4 (8.70)	

Abbreviations: ASD, atrial septal defect; IQR, interquartile range; PDA, patent ductus arteriosus; PFO, patent foramen ovale; VSD, ventricular septal defect.

^a^ Data are expressed as No. (%) unless otherwise indicated.

^b^ *p* values are provided by the Chi‐square test or Fisher's exact test, or Kruskal‐Wallis test in ordered categorical variables and onset age.

^c^ The duration and frequency of seizures were recorded after treatment with anti‐seizure medications (ASMs).

### 3.3 Sensitivity Analyses

Using the actual age as the exposure duration, Poisson regression showed that PFO was significantly associated with an increased risk of epilepsy both in the overall patients with (RR = 2.86, 95% CI: 1.75–4.71) and without (RR = 2.31, 95% CI: 1.16–5.11) PFO closure surgery (Table S2), which is consistent with the results of the main analysis. In another sensitivity analysis which excluded the patients with a short follow‐up (< half a year), the results also remained similar (Table S3).

## 4 Discussion

This cohort study, for the first time, demonstrated that patients with long‐term exposure to PFO have a 3.28 times risk of developing epilepsy than those with ASD, PDA, or VSD after adjusting for confounding factors such as age, stroke, and hypoxia at birth. This risk appears to be reduced by performing PFO closure surgery. These findings suggest that PFO may be an independent risk factor for epilepsy and that PFO closure procedures are related to a lower risk of epilepsy.

The physiological mechanism of congenital heart disease‐induced epilepsy is still mostly unknown, but the mechanisms that are currently accepted indicate that vasoactive substances can be eliminated or metabolized through the pulmonary circulation [31]. However, congenital heart malformations may cause venous blood to shunt and not circulate in the lungs, mixing it with arterial blood. As a result, chemicals and hormones like serotonin may traverse the blood–brain barrier and avoid pulmonary circulation, which can lead to the development of epilepsy [17].

Furthermore, a genetic investigation has demonstrated that the comorbidity of congenital cardiac disease and epilepsy can be explained by the voltage‐gated calcium channel (VGCC) system. A theoretical foundation for epilepsy brought on by congenital heart dysfunction was established by these investigations [32].

Most previous studies focused only on the overall incidence of epilepsy in patients with CHD, particularly those with complex CHD. A Danish cohort study involving 15,222 patients with CHD described the overall cumulative incidence rate of epilepsy in CHD patients aged 15 years to be 5%, which was 3.7 times that in the general population before 5 years of age, and 2.3 times that from 5 to 32 years of age [13]. A nationwide cohort study in Sweden showed that the cumulative incidence of epilepsy in the study period was 3% in patients with CHD and 0.9% in general population. The risk of epilepsy was 3.6 times higher (95% CI: 3.4–3.8) in patients with CHD than in controls [17]. A cross‐sectional study in the UK also showed a prevalence of epilepsy in patients with CHD of 2.4%, approximately 2.4 times that in patients without CHD (prevalence of epilepsy in general population, 0.9%) [31]. These studies revealed that complex CHD is an important factor explaining the significantly higher IR in epilepsy compared to that in the general population. Our findings fill a gap in this field by providing evidence on the incidence of epilepsy in patients with simple CHD compared among different types of simple CHD. We found that the incidence of epilepsy in PFO patients was significantly higher than that in ASD patients or PDA patients. Although we did not have general population data in this study, a previous study showed that the age‐standardized incidence of epilepsy in the general population in China was 0.246/1000 person‐years, which was significantly lower than the age‐standardized incidence of epilepsy (1.29/1000 person‐years) in PFO patients of this study, supporting that PFO was a risk factor of epilepsy [33].

**FIGURE 2:**
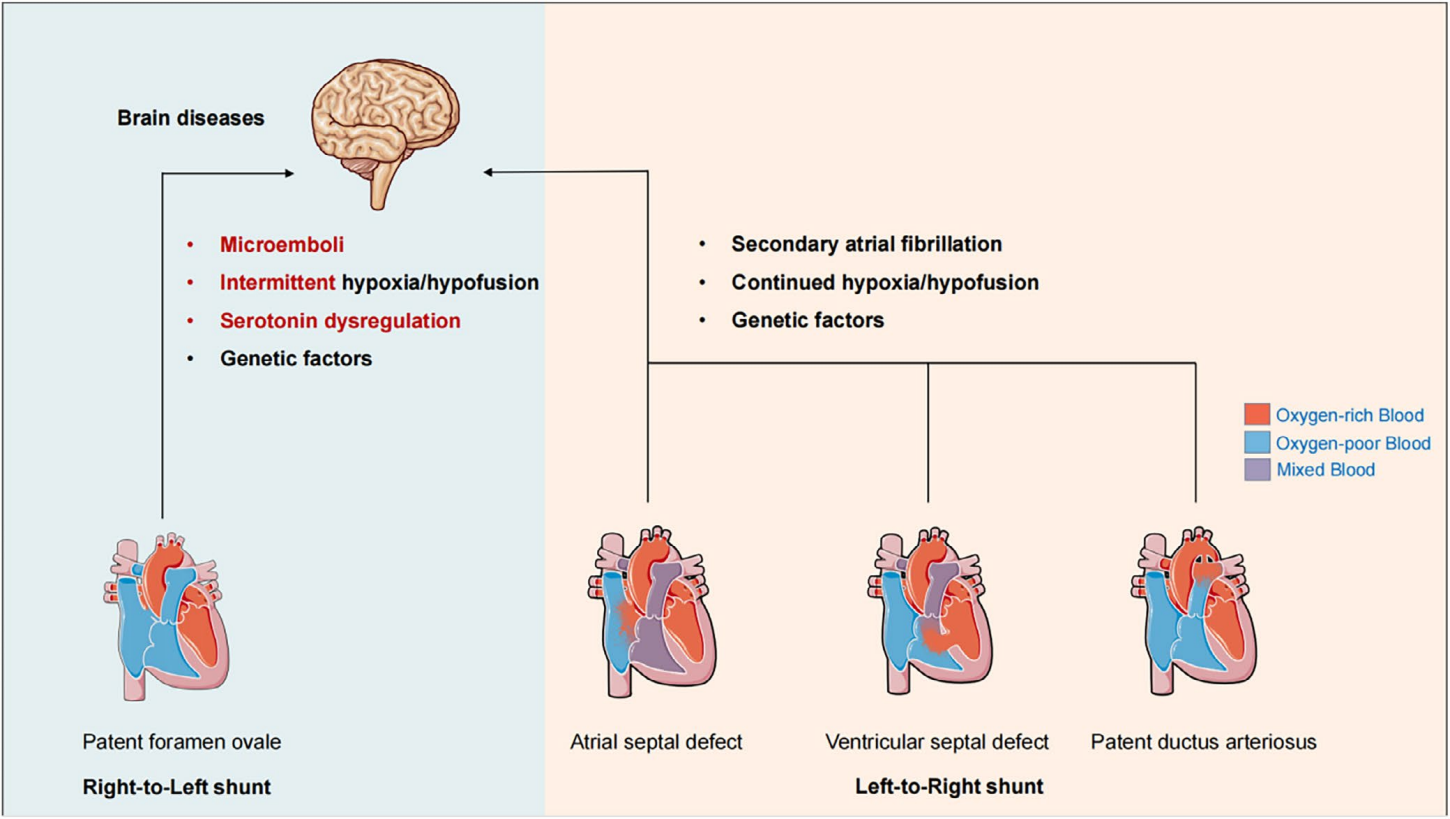
Mechanism hypothesis of the association between simple congenital heart diseases and the central nervous system of the brain.

The association between PFO and the incidence of epilepsy, as well as the potential efficacy of PFO closure surgery, suggest that the mechanisms by which PFO affects cerebral neurological function may differ from those of other simple CHD types. Several possible mechanisms may explain this phenomenon, as shown in Figure 2. First, in contrast with the hemodynamic changes observed in the other three types of simple CHD, which mostly show a continuous left‐to‐right shunt, PFO has a distinct anatomical structure and exhibits a diametric clinical manifestation (paroxysmal right‐to‐left shunt) [17, 27]. Therefore, we hypothesized that PFO causes cerebral neurological dysfunction by allowing paradoxical embolism, inflammatory mediators, choline compounds, glutamine, and unoxygenated venous blood to bypass the pulmonary circulation and directly enter the systemic circulation or even the brain [34]. These mechanisms could cause cerebral hypoxia, cerebral vascular endothelial cell dysfunction, or cortical excitability changes, which in turn may increase the risk of stroke, migraine, and obstructive sleep apnea (OSA) [35, 36]. A previous study showed a significant decrease in partial oxygen pressure in PWE accompanied by a right‐to‐left shunt, indicating a potential hypoxia mechanism [37].

In addition, vasoactive substances and hypoxia can trigger cortical spreading inhibition (CSD) [38, 39, 40], which can increase the excitability of neurons and lead to epileptiform discharges in partially suppressed neuronal tissues [41]. CSD can also increase the recurrence rate and amplitude of spontaneous abnormal epileptiform discharges in human hippocampal slices [42]. Simultaneously, CSD activates neuronal Pannexin1 channel, which stimulates neuronal hyperactivity during seizures [43].

If PFO is determined to be a potential cause of epilepsy in future observational studies with larger samples, PFO screening will be a worthwhile clinical diagnostic tool for PWE. In addition, if PFO closure therapy is further shown to reduce epileptic seizures in randomized clinical trials, it may become an alternative method for doctors to consider in the treatment of epileptic seizures that might provide additional health benefits to affected patients.

However, several limitations must be acknowledged. First, although our study had a large sample population, the low incidence of epilepsy was a limitation because it limited the statistical power in many of the described analyses, such as the test for interaction between CHD and CHD surgery. In the future, we aim to verify our findings using a greatly expanded sample size. Second, our study lacked data on patients' behavior and lifestyle. Previously, some studies found that children with simple CHD or complex CHD have increased risk for poor motor function and secondary lifestyle, and those factors were also associated with epileptic seizures. Thus, there could be residual confounding caused by those factors were in our study [44, 45].

In addition, although we found that CHD surgery, especially PFO closure procedure, was related to a lower risk of epilepsy, further rigorous studies such as randomized clinical trials are needed to better evaluate the effect of CHD surgical procedures in reducing the risk of epilepsy, and the mechanism for the association between simple CHD and epilepsy remains to be investigated [46]. Finally, as our study was based on hospital data rather than general population survey, the incidence of epilepsy in non‐CHD patients was not available. Thus, we could not compare the CHD and non‐CHD patients within‐study directly, which limited our ability in assessing the etiological role of CHD in the development of epilepsy. Further studies with general population data are warranted.

## 5 Conclusions

PFO is associated with a 3.28 times risk of epilepsy when compared with ASD, and PFO closure procedure is related to a lower risk of epilepsy. These findings have implications for future research on the pathogenesis of PFO and incidence of central nervous system diseases, and perhaps importantly, for the prevention and treatment of epilepsy in patients with PFO.

## Author Contributions

L.C. designed the study. T.S., Y.C., Y.T., Y.L., and H.L. retrieved, collected, anonymized, curated, and verified the data. Z.Y. and S.J. analyzed the data, interpreted the results, and produced the figures. The manuscript was written by all authors and reviewed by L.C., Z.Y., and S.J. All authors had full access to all the data in the study and had final responsibility for the decision to submit for publication.

## Ethics Statement

We confirm that we have read the Journal's position on issues involved in ethical publication and affirm that this report is consistent with those guidelines. This study had received ethical approval from the Ethics Committee of West China Hospital of Sichuan University and registered on the Chinese Clinical Trial Registry (Registration number: ChiCTR2100052710). All participants were required to sign informed consent.

## Conflicts of Interest

The authors declare no conflicts of interest.

## Supporting information


Supporting Information S1.

Supporting Information S2.

## Data Availability

Due to the nature of this research using data from the hospital's case management system, participants of this study did not agree for their data to be shared publicly, so Supporting Information S1 and S2 are not available.
