# Supplementary material for: Comparing the Risk of Epilepsy in Patients With Simple Congenital Heart Diseases: A Prospective Cohort Study
Source: CNS Neurosci Ther. 2025 Feb 7;31(2):e70230. doi: 10.1111/cns.70230 (PMC11803515; doi:10.1111/cns.70230)
Supplement: Supplementary file 1 — Supporting Information S1. [file CNS-31-e70230-s002.docx]

**Supplementary materials - Sup.1**

This supplementary material introduced the diagnostic procedures and methods for simple congenital heart diseases (CHD) and epilepsy, the mainly examination methods including echocardiography, electrocardiogram (ECG), cardiac magnetic resonance (CMR) imaging, computed tomography (CT), electroencephalography (EEG), and magnetic resonance imaging (MRI), as shown in part 1 (Diagnostic process of simple congenital heart diseases and epilepsy). The second section (part 2) presented the inclusion of patients with simple CHD in different years and the results of sensitivity analysis. The third part showed the normality test results of continuous variables and their distribution in different types of simple CHD patients.

1. **Diagnostic process of simple congenital heart diseases and epilepsy**

**1.1 Diagnosis of simple congenital heart diseases**

The diagnostic process of simple congenital heart diseases mainly depends on the results of imaging examination, and assisted by clinical examination and electrocardiograph (ECG) (Clinical examination plays a major role and includes careful evaluation of any changes in auscultation findings, blood pressure, or development of signs of heart failure, ECG plays a role in diagnosing whether a patient is accompanied by arrhythmia or heart hypertrophy).

Among the imaging methods, echocardiography is the most important first-line imaging method, and cardiovascular magnetic resonance (CMR) imaging and cardiovascular computed tomography (CCT) are auxiliary examination methods, which are mainly suitable for preoperative examination before congenital heart surgery.

The diagnosis of PFO was conformed by the American Society of Echocardiography and Society for Cardiac Angiography and Intervention (ASE and SCAI, 2015 version). Patients who underwent PFO closure were required to have at least a secondary or higher grade of RLS volume, which was detected using contrast transthoracic echocardiography (cTTE) and the observation of shunting microbubbles following contrast injection. For semiquantitative assessment, the RLS volume detected by TTE is categorized into four grades based on the number of microvesicles detected per frame in the left atrium: grade 0 = no microbubbles present; grade I = 1 - 10 microbubbles; grade II = 11 - 30 microbubbles; grade III = over 30 microbubbles, or left atrium almost filled with microbubbles, or left atrial opacity. If the RLS volume of PFO patients exceeded grade I, individuals were advised to undergo transesophageal echocardiography (cTEE).

**1.2 Diagnosis of epilepsy**

Epilepsy was confirmed using the 2014 International League Against Epilepsy (ILAE) standards. The diagnosis of epilepsy was made by two neurologists on the basis of clinical seizure findings, electroencephalography (EEG), and magnetic resonance imaging (MRI).

The identified cases of drug-resistant epilepsy based on the ILAE definition of drug resistant epilepsy (2010 version), which required the epilepsy patient to comply with the failure of adequate trials of two tolerated, appropriately chosen and used antiepileptic drug schedules (whether as monotherapies or in combination) to achieve sustained seizure freedom. In our hospital, we confirmed patients with drug-resistant epilepsy who had failed to effectively control their seizures after taking two ASMs for at least more than one year, the diagnostic process was determined jointly by two experienced neurologists, and the diagnostic process also involved assessing seizure frequency and symptoms, as well as reviewing EEG and MRI results.

**1.3 Echocardiography**

In most instances, echocardiography provides information on basic cardiac anatomy, including the orientation and position of the heart, venous return, connection of the atria and ventricles, and origin of the great arteries. It enables the evaluation of the morphology of the cardiac chambers, ventricular function, and detection and evaluation of shunt lesions, as well as the morphology and function of the heart valves. Assessments of ventricular volume overload (increase in end-diastolic volume and stroke volume) and pressure overload (hypertrophy and increase in ventricular pressure) are of major importance. Doppler echocardiographic information includes hemodynamic data, such as gradients across obstructions and right ventricle (RV) pressure/pulmonary artery pressure (PAP) (obtained from tricuspid regurgitation [TR] velocity), as well as flow calculations. Although echocardiography can provide comprehensive information, it is highly user-dependent and requires special expertise in patients with grown-up congenital heart disease (GUCH); its limitations must also be taken into consideration. The assessment of ventricular volume and function may be complicated by geometry and regional incoordination, particularly in systemic and non-systemic RVs or univentricular hearts (UVHs). Doppler gradients may be misleading sometimes, particularly in RV outflow tract obstruction (RVOTO), coarctation of the aorta (CoA), and stenoses in series. Furthermore, venous return and the great arteries may be difficult to visualize.

All the patients were examined in detail using transthoracic echocardiography (Philips IE 33 with 1–5 MHz or 3–8 MHz multiplane transducers) in the long-axis view of the parasternal left ventricle (LV), short-axis view of the great artery, four-chamber view of the apex, five-chamber view of the apex, aortic arch view of the superior sternal fossa, four-chamber view of the inferior xiphoid process, and two-chamber view of the inferior xiphoid process.

**1.4 Cardiac magnetic resonance (CMR) imaging**

In clinical practice, there are several indications for CMR when assessing simple CHD in adults, including:

1. as an alternative to echocardiography when both techniques provide similar information; however, echocardiography cannot achieve sufficient quality. Most information described in Section 3.2.1 can also be obtained by CMR, although echocardiography is superior in estimating gradients and PAP and in detecting small, highly mobile structures such as vegetations.
2. As the second method when echocardiography measurements are borderline or ambiguous. LV volumes and LV ejection fraction (LVEF) may be critical in the management of patients with GUCH (particularly in the context of volume overload), and the same applies to the quantification in valvular regurgitation. When the values from Doppler echocardiography are borderline or ambiguous, CMR should be applied as a secondary method to corroborate or dismiss the echocardiographic values before making clinical decisions.
3. Instances where CMR is considered superior to echocardiography, and should be regularly used when the information is essential for patient management.

**1.5 Cardiovascular computed tomography (CCT)**

CCT plays an increasingly important role in the imaging of patients with GUCH, providing excellent spatial resolution and has a rapid acquisition time. It is particularly useful for imaging epicardial coronary arteries and collateral arteries and for parenchymal lung disease. Ventricular size and function can be assessed with inferior temporal resolution, compared with CMR. The major drawback of most current CCT systems is the high dose of ionizing radiation, which makes serial use unattractive. CCT is currently more widely available than CMR, and thus plays a role in acute situations. Moreover, recent developments, such as ECG-triggered acquisition and newer rotational techniques, have substantially reduced the amount of radiation, which may make CCT a more attractive alternative to CMR in the coming years.

**1.6 Contrast transthoracic echocardiography (cTTE)**

For patients suspected clinically of having a PFO, cTTE was additionally used for diagnosis. A microbubble bolus from an agitated solution of 8 mL saline, 1 mL blood, and 1 mL air was injected into the antecubital veins to increase sensitivity. Prior to the examination, sonographers informed the participants about the Valsalva maneuver. Participants were assessed for PFO at rest and during provocative maneuvers (Valsalva maneuver and while coughing). The presence of a PFO was considered positive if microbubbles were present in the left atrium or LV within three cardiac cycles of the maximum right atrial opacification.

**1.7 Electroencephalography (EEG)**

EEG is a specific investigation that supports the diagnosis of epilepsy by demonstrating interictal epileptiform activity in most individuals with epilepsy. EEG can also help classify epilepsy as focal or generalized, and can suggest certain epileptic syndromes. However, epileptiform activity is absent in the first EEG in approximately half of affected individuals, and the interictal EEG remains normal in approximately 10% of affected individuals. Prolonged EEG recordings increase the odds of detecting epileptiform activity and provide the opportunity to capture ictal discharges for a more definitive diagnosis and classification.

Video-EEG (V-EEG) also allows the correlation of clinical and EEG abnormalities and the analysis of seizure semiology. V-EEG has become an essential element of presurgical evaluation for the localization of the epileptogenic zone; however, confidence in the EEG data requires congruence with other presurgical tests, particularly MRI. Interictal slow activity defines a functional deficit zone, interictal epileptiform discharges define the irritative zone, ictal EEG onset defines the ictal onset zone, and analysis of seizure semiology on video helps define the symptomatogenic zone. The EEG pattern and frequency at the ictal onset can improve the localization and may help predict seizure outcomes. EEG is also crucial in the diagnosis of nonconvulsive status epilepticus.

**1.8 Magnetic resonance imaging (MRI)**

All patients with intractable epilepsy considered for surgery should undergo high-resolution structural MRI because the success of operative treatment is directly related to the ability to precisely localize the region of seizure onset and underlying structural abnormalities. Since a structural abnormality does not necessarily indicate the site of seizure origin, clinical, EEG, and other data must be correlated with imaging. MR-based functional imaging methods such as positron emission tomography (PET) and single-photon emission CT (SPECT) can provide additional information and assist in generating hypotheses for invasive EEG if structural imaging is normal or equivocal, shows diffuse abnormalities, or if there is discordance between different modalities. Postoperative MRI is useful for identifying the extent of cortical resection or the presence of residual pathology, particularly if seizures persist after surgery.

The sensitivity of MRI for detecting abnormalities depends on the pathological substrate, MRI techniques applied, and experience of the interpreting physician. An optimal routine MRI protocol should include T1- and T2-weighted, proton density and fluid-attenuated inversion recovery (FLAIR) sequences. These contrasts must be acquired in at least two orthogonal planes covering the whole brain using the minimum possible slice thickness. An oblique coronal plane oriented perpendicular to the long axis of the hippocampus provides the best definition of the medial temporal lobe structures. In general, T1-weighted images give the best definition of the anatomy and differentiate gray and white matter, while T2-weighted images provide high sensitivity for detecting brain pathology. A three dimensional T1-weighted volume sequence with a partition size of 1.5 mm or less should be included because these images may be reformatted in any orientation and used for post-acquisition processing, such as in measuring hippocampal volumes. FLAIR imaging produces heavy T2-weighting and suppresses cerebrospinal fluid (CSF) signals. This provides high lesion contrast in areas close to the CSF and enables anatomical detail to be observed with greater visibility than with conventional T2-weighted sequences. Gadolinium does not improve the sensitivity of MRI in patients with epilepsy, but may be useful for characterizing intracerebral lesions associated with the breakdown of the blood brain barrier.

Visual assessment of all images should be performed in addition to the knowledge of the clinical situation, by a neuroimaging specialist with expertise in epilepsy imaging. In addition to the identification of obvious lesions, a systematic approach to image reporting is essential because dual pathology is common. It is important to search for subtle cortical abnormalities, including focal atrophic abnormalities and dysplastic lesions without mass effects, and to evaluate the hippocampus regardless of other MR findings. Thick image slices, rotated orientation, and the presence of normal anatomical variations make visual assessment more difficult and increase the risk of misdiagnosis.

**1.9 Electrocardiogram**

The electrocardiogram (ECG) is a non-invasive diagnostic tool used to assess the heart's electrical activity by placing electrodes on the body to record signals from different angles (12-lead system). The process involves preparing the patient, capturing electrical signals, and analyzing waveforms such as the P wave, QRS complex, and T wave to identify abnormalities in heart rhythm, conduction, and ischemic changes. ECG is essential in diagnosing cardiovascular conditions like myocardial infarction, arrhythmias, and heart blocks, and is used in pre-operative evaluations, emergency chest pain assessments, chronic disease monitoring, and athlete screenings. Its applications extend to continuous monitoring (e.g., Holter monitoring), stress testing, and advanced remote telemetry, making it vital for both clinical practice and research.

1. **Simple congenital heart diseases cohort follow-up information and the results of sensitivity analysis**

**Sup. Table 1 The frequency and follow-up period of patients with each types of simple congenital heart diseases enrolled in different yearly intervals**

|  | **Total cases** | **Classification of simple congenital heart diseases ^a^** | | | |
| --- | --- | --- | --- | --- | --- |
|  |  | **ASD (N = 4985)** | **PDA (N = 1432)** | **VSD (N = 2362)** | **PFO (N = 2135)** |
| **Follow-up patients Num of total (percentage)** | |  |  |  |  |
| **Enrolled in 2010 and before** | 676 | 299 (44.23) | 84 (12.43) | 107 (15.83) | 186 (27.51) |
| **Enrolled between 2011 and 2012** | 2221 | 1039 (46.78) | 307 (13.82) | 675 (30.39) | 200 (9.01) |
| **Enrolled between 2013 and 2014** | 2580 | 1221 (47.33) | 313 (12.13) | 629 (24.38) | 417 (16.16) |
| **Enrolled between 2015 and 2016** | 2497 | 1193 (47.78) | 305 (12.21) | 508 (20.34) | 491 (19.66) |
| **Enrolled between 2017 and 2018** | 2001 | 843 (42.13) | 284 (14.19) | 340 (16.99) | 534 (26.69) |
| **Enrolled in 2019 and after** | 939 | 390 (41.53) | 139 (14.80) | 103 (10.97) | 307 (32.69) |
| **Sum enrolled cases** | 10914 | 4985 (45.68) | 1432 (13.12) | 2362 (21.64) | 2135 (19.56) |
| **Follow-up time, mean year (min - max)** | |  |  |  |  |
| **Enrolled in 2010 and before** | 8.66 (0.01 - 13.25) | 8.76 (0.01 - 13.25) | 8.48 (0.02 - 12.96) | 6.31 (0.01 - 13.04) | 9.94 (1.19 - 13.17) |
| **Enrolled between 2011 and 2012** | 2.07 (0.01 - 10.15) | 2.28 (0.01 - 10.08) | 1.85 (0.01 - 10.15) | 1.43 (0.01 - 9.75) | 3.5 (0.01 - 10.08) |
| **Enrolled between 2013 and 2014** | 2.38 (0.01 - 8.26) | 2.46 (0.01 - 8.21) | 2.15 (0.01 - 7.83) | 2.09 (0.01 - 8.05) | 2.74 (0.01 - 8.26) |
| **Enrolled between 2015 and 2016** | 1.77 (0.01 - 6.26) | 1.81 (0.01 - 6.26) | 1.47 (0.01 - 6.03) | 1.79 (0.01 - 6.1) | 1.84 (0.01 - 6.16) |
| **Enrolled between 2017 and 2018** | 1.12 (0.01 - 4.26) | 1.15 (0.01 - 4.26) | 0.97 (0.01 - 3.92) | 1.15 (0.01 - 4.07) | 1.12 (0.01 - 4.16) |
| **Enrolled in 2019 and after** | 0.65 (0.01 - 2.29) | 0.70 (0.01- 2.29) | 0.67 (0.01 - 2.05) | 0.80 (0.01 - 2.19) | 0.53 (0.01 - 2.23) |
| **Sum enrolled cases** | 2.19 (0.01 - 13.25) | 2.29 (0.01 - 13.25) | 1.93 (0.01 - 12.96) | 1.83 (0.01 - 13.04) | 2.51 (0.01 - 13.17) |
| **Epilepsy screening, Num of total** | |  |  |  |  |
| **Response for having seizures** | 342 | 103 | 41 | 55 | 143 |
| **Receiving hospital diagnosis** | 270 | 86 | 30 | 46 | 108 |
| Abbreviation: ASD, atrial septal defect; PDA, patent ductus arteriosus; VSD, ventricular septal defect; PFO, patent foramen ovale  ^a^ Data are expressed as No. (%) unless otherwise indicated. | | | | | |

**Sup. Table 2 The risk ratio, incidence rate, and incidence rate difference of** **epilepsy in patients with simple congenital heart diseases using age as exposure duration**

|  | **Person-years** | **No. of CHD cases** | **No. of epilepsy** | **Adjusted risk ratio**  **(95% CI)^a^** | **Incidence rate**  **/ 1000 person-years** | **Incidence rate difference**  **/ 1000 person-years** | **Adjusted incidence rate**  **/ 1000 person-years** |
| --- | --- | --- | --- | --- | --- | --- | --- |
| **Classification of simple CHD** | | |  |  |  |  |  |
| ASD | 196537.00 | 4985 | 30 | 1 [Reference] | 0.15 | 0 [Reference] | 0.15 |
| PDA | 44694.00 | 1432 | 11 | 1.47 (0.70 to 2.86) | 0.25 | 0.09 (-0.06 to 0.25) | 0.37 |
| VSD | 50443.00 | 2362 | 21 | 2.50 (1.41 to 4.36) | 0.42 | 0.26 (0.08 to 0.45) | 1.05 |
| PFO | 103027.00 | 2135 | 46 | 2.86 (1.75 to 4.71) | 0.45 | 0.29 (0.15 to 0.43) | 1.29 |
| **Simple CHD with surgery history** | | |  |  |  |  |  |
| ASD | 151573.00 | 3959 | 21 | 1 [Reference] | 0.14 | 0 [Reference] | 0.14 |
| PDA | 35000.00 | 1145 | 8 | 1.64 (0.67 to 3.62) | 0.23 | 0.09 (-0.08 to 0.26) | 0.38 |
| VSD | 35539.00 | 1892 | 15 | 2.46 (1.21 to 4.91) | 0.42 | 0.28 (0.06 to 0.51) | 1.03 |
| PFO | 13989.00 | 281 | 5 | 1.72 (0.52 to 4.76) | 0.36 | 0.22 (-0.10 to 0.54) | 0.62 |
| **Simple CHD without a surgery history** | | |  |  |  |  |  |
| ASD | 44964.00 | 1026 | 9 | 1 [Reference] | 0.20 | 0 [Reference] | 0.20 |
| PDA | 9694.00 | 287 | 3 | 1.30 (0.29 to 4.36) | 0.31 | 0.11 (-0.26 to 0.48) | 0.40 |
| VSD | 14904.00 | 470 | 6 | 2.12 (0.70 to 5.92) | 0.40 | 0.20 (-0.15 to 0.55) | 0.85 |
| PFO | 89038.00 | 1854 | 41 | 2.31 (1.16 to 5.11) | 0.46 | 0.26 (0.07 to 0.45) | 1.06 |
| Abbreviation: ASD, atrial septal defect; PDA, patent ductus arteriosus; VSD, ventricular septal defect; PFO, patent foramen ovale; CI, confidence interval  ^a^ The risk ratio was adjusted for gender, minority, smoking, alcohol, hypertension, diabetes, stroke, migraine, birth hypoxia, birth preterm, febrile convulsion, congenital anomaly. | | | | | | | |

**Sup. Table 3 Sensitivity analysis of the risk ratio of epilepsy in patients with simple congenital heart diseases followed up for more than half a year**

| **Categories of simple CHD** | **Risk ratio of epilepsy** | | |
| --- | --- | --- | --- |
|  | **Total (95% CI)** | **With surgery history**  **(95% CI)** | **Without surgery history**  **(95% CI)** |
| ASD | 1 [Reference] | 1 [Reference] | 1 [Reference] |
| PDA | 1.70 (0.77 to 3.45) | 1.63 (0.62 to 3.85) | 1.60 (0.34 to 5.80) |
| VSD | 1.87 (0.95 to 3.61) | 1.71 (0.71 to 3.99) | 1.94 (0.60 to 6.09) |
| PFO | 3.31 (1.91 to 5.80) | 1.70 (0.43 to 5.26) | 2.60 (1.19 to 6.52) |
| Abbreviation: ASD, atrial septal defect; PDA, patent ductus arteriosus; VSD, ventricular septal defect; PFO, patent foramen ovale; CI, confidence interval | | | |

1. **The normality test results of continuous variables and their distribution in different types of simple CHD patients**

In our study, the age of all recruited patients with simple CHD as well as the onset age of epilepsy patients were continuous variables, so normality was tested for all of them, and the results were shown in Figures 1 and 2 and Table 4. Furthermore, we also showed the distribution of these two variables in patients with different types of simple CHD, as shown in Figures 3 and 4.

**
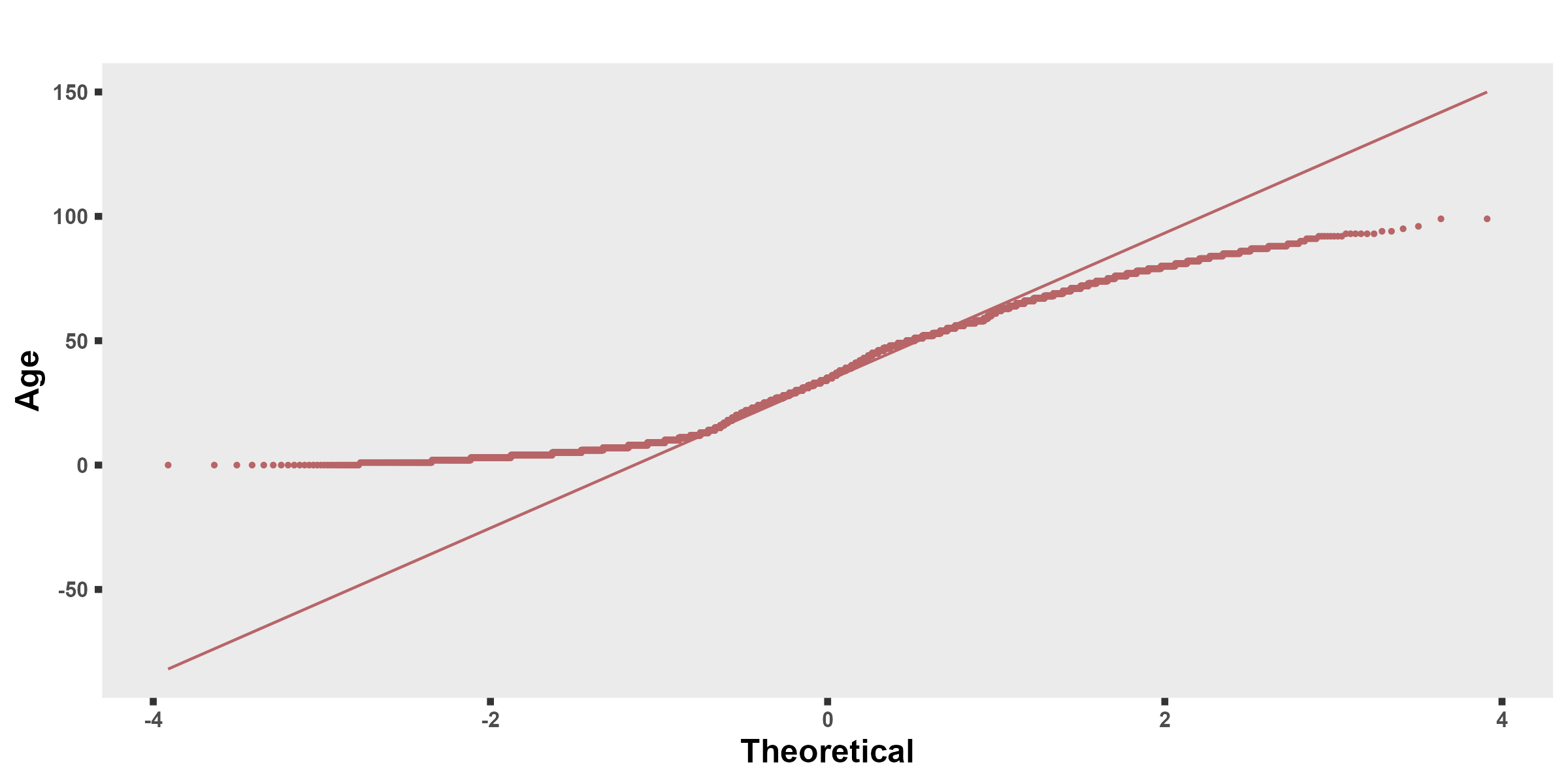
**

**Sup. Figure 1 The quantile-quantile (Q-Q) plots of the age in patients with simple congenital heart diseases**

**
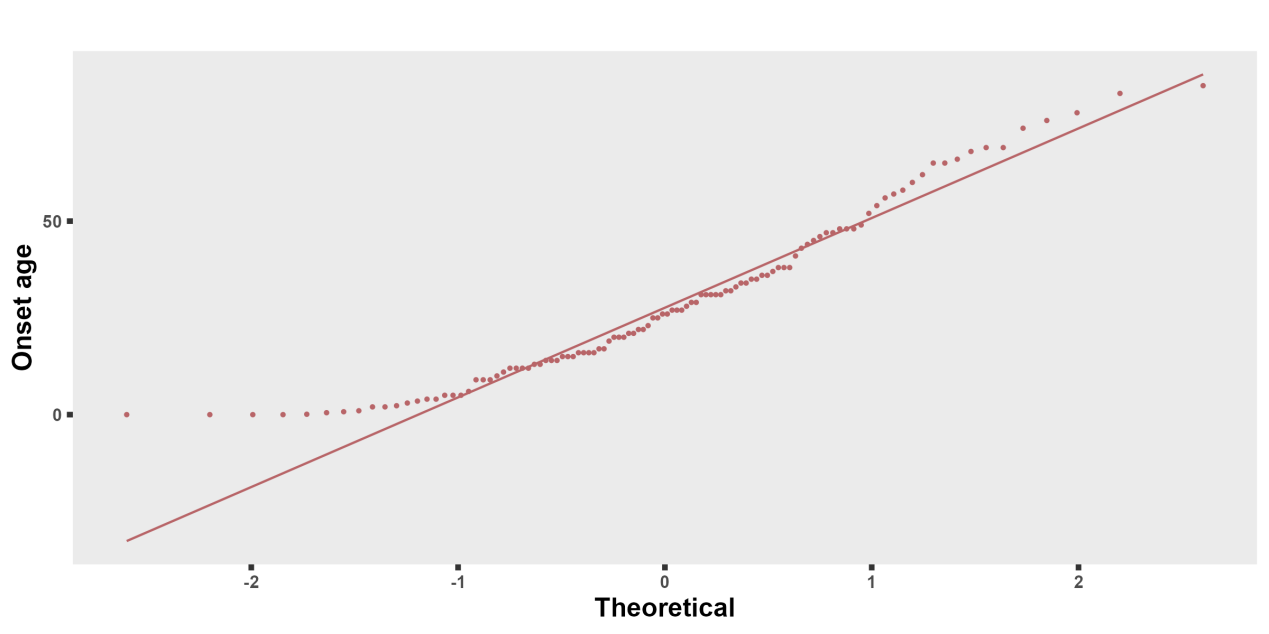
**

**Sup. Figure 2 The quantile-quantile (Q-Q) plots of the onset age in patients with epilepsy and simple congenital heart diseases**

**Sup.Table 4 The normality test results of continuous variables in patients with simple CHD**

| **Variables** | **Shapiro-Wilk normality test** | | **Kolmogorov-Smirnov normality test** | |
| --- | --- | --- | --- | --- |
|  | **W value** | ***p* value** | **D value** | ***p* value** |
| Age | 0.958 | < .001 | 0.088 | < .001 |
| Onset age of epilepsy | 0.940 | < .001 | 0.097 | 0.263 |

**
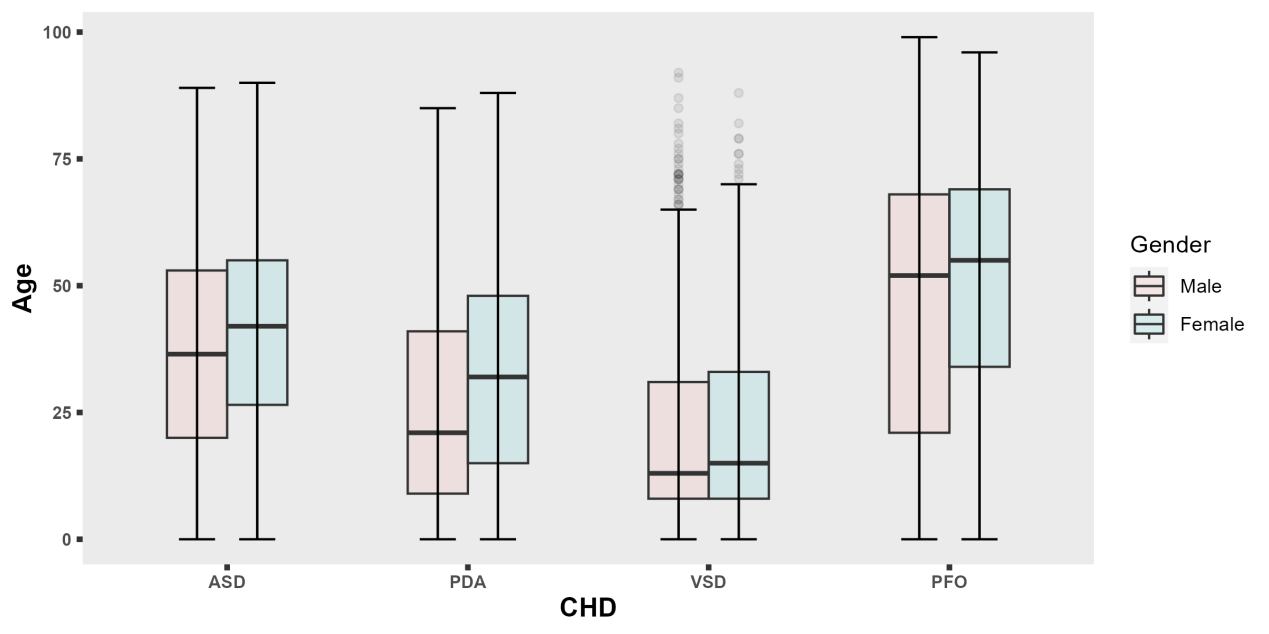
**

**Sup.Figure 3 The distribution of the age in patients with simple congenital heart diseases (In male patients with simple CHD, the mean ± SD of age for each type of CHD respectively was: ASD = 37.070 ± 21.093, PDA = 26.899 ± 20.642, VSD = 20.897 ± 17.383, PFO = 46.046 ± 27.260; in female patients with simple CHD, the mean ± SD of age for each type of CHD respectively was: ASD = 40.562 ± 19.030, PDA = 32.648 ± 19.428, VSD = 21.894 ± 17.183, PFO = 50.343 ± 25.131)**

**
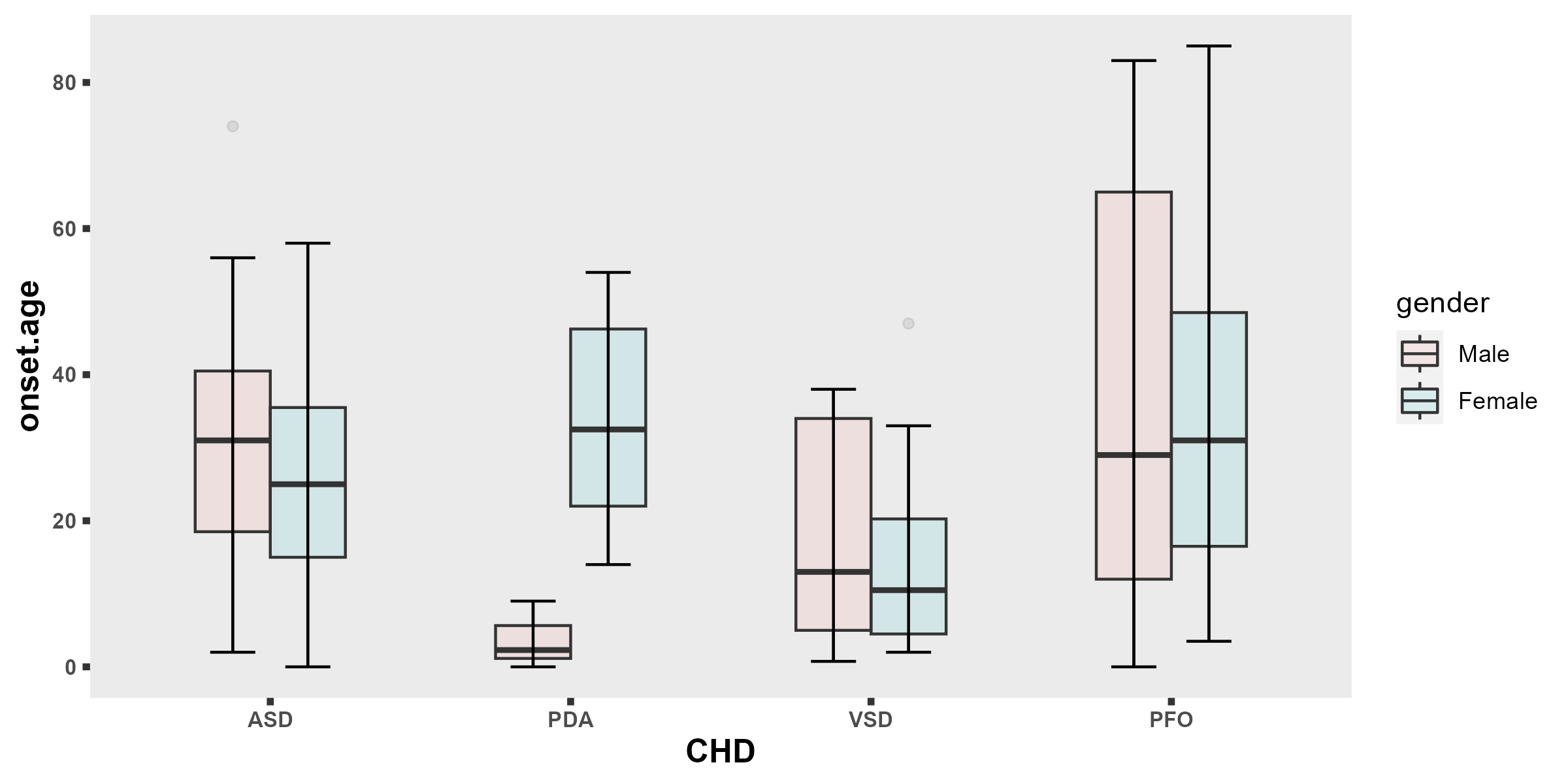
**

**Sup.Figure 4 The distribution of the onset age in patients with epilepsy and simple congenital heart diseases (In male patients with simple CHD and epilepsy, the mean ± SD of onset age for each type of CHD respectively was: ASD = 31.909 ± 20.647, PDA = 3.767 ± 4.676, VSD = 17.442 ± 14.200, PFO = 36.591 ± 28.357; in female patients with simple CHD and epilepsy, the mean ± SD of onset age for each type of CHD respectively was: ASD = 26.158 ± 16.463, PDA = 33.500 ± 14.745, VSD = 15.875 ± 16.040, PFO = 34.848 ± 21.983)**
