# Supplementary material for: Comparing the Risk of Epilepsy in Patients With Simple Congenital Heart Diseases: A Prospective Cohort Study
Source: CNS Neurosci Ther. 2025 Feb 7;31(2):e70230. doi: 10.1111/cns.70230 (PMC11803515; doi:10.1111/cns.70230)
Supplement: Supplementary file 2 — Supporting Information S2. [file CNS-31-e70230-s001.docx]

**Supplementary materials - Sup.2**

**Reporting checklist for cohort study. Based on the STROBE cohort guidelines.**

| **Var** | **No.** | **Reporting Items** | **Page Number** |
| --- | --- | --- | --- |
| **Title and Abstract** |  |  |  |
| Title | # 1a | Indicate the study’s design with a commonly used term in the title or the abstract. | **Page 01, lines 001 - 002** |
| Abstract | # 1b | Provide in the abstract an informative and balanced summary of what was done and what was found. | **Page 01, lines 023 - 050** |
| **Introduction** |  |  |  |
| Background / rationale | # 02 | Explain the scientific background and rationale for the investigation being reported. | **Page 04, lines 091 - 122** |
| Objectives | # 03 | State specific objectives, including any prespecified hypotheses. | **Page 05, lines 122 - 124** |
| **Methods** |  |  |  |
| Study design | # 04 | Present key elements of study design early in the paper. | **Page 05, lines 126 - 136** |
| Setting | # 05 | Describe the setting, locations, and relevant dates, including periods of recruitment, exposure, follow-up, and data collection. | **Page 06, lines 150 - 187** |
| Eligibility criteria | # 6a | Give the eligibility criteria, and the sources and methods of selection of participants. Describe methods of follow-up. | **Page 06, lines 150 - 187** |
| Eligibility criteria | # 6b | For matched studies, give matching criteria and number of exposed and unexposed. | **N/A** |
| Variables | # 07 | Clearly define all outcomes, exposures, predictors, potential confounders, and effect modifiers. Give diagnostic criteria, if applicable. | **Page 06, lines 150 - 159** |
| Data sources / measurement | # 08 | For each variable of interest give sources of data and details of methods of assessment (measurement).  Describe comparability of assessment methods if there is more than one group. Give information separately for exposed and unexposed groups if applicable. | **Page 05, lines 126 - 136** |
| Bias | # 09 | Describe any efforts to address potential sources of bias. | **Page 05, lines 131 - 136** |
| Study size | # 10 | Explain how the study size was arrived at. | **Page 07, lines 231 - 233** |
| Quantitative variables | # 11 | Explain how quantitative variables were handled in the analyses. If applicable, describe which groupings were chosen, and why. | **Page 07, lines 189 - 198** |
| Statistical methods | # 12a | Describe all statistical methods, including those used to control for confounding. | **Page 07, lines 199 - 220** |
| Statistical methods | # 12b | Describe any methods used to examine subgroups and interactions. | **Page 07, lines 210 - 215** |
| Statistical methods | # 12c | Explain how missing data were addressed. | **Page 07, lines 189 - 190** |
| Statistical methods | # 12d | If applicable, explain how loss to follow-up was addressed. | **Page 06, lines 161 - 164** |
| Statistical methods | # 12e | Describe any sensitivity analyses. | **Page 07, lines 217 - 218** |
| **Results** |  |  |  |
| Participants | # 13a | Report numbers of individuals at each stage of study - eg numbers potentially eligible, examined for eligibility, confirmed eligible, included in the study, completing follow-up, and analyzed.  Give information separately for exposed and unexposed groups if applicable. | **Page 09, lines 234 - 247** |
| Participants | # 13b | Give reasons for non-participation at each stage. | **N/A** |
| Participants | # 13c | Consider use of a flow diagram. | **Figure 1** |
| Descriptive data | # 14a | Give characteristics of study participants (eg demographic, clinical, social) and information on exposures and potential confounders.  Give information separately for exposed and unexposed groups if applicable. | **Page 09, lines 234 - 247** |
| Descriptive data | # 14b | Indicate number of participants with missing data for each variable of interest. | **N/A** |
| Descriptive data | # 14c | Summarize follow-up time (eg, average and total amount). | **Page 08, lines 233 - 234** |
| Outcome data | # 15 | Report numbers of outcome events or summary measures over time.  Give information separately for exposed and unexposed groups if applicable. | **Page 09, lines 248 - 260** |
| Main results | # 16a | Give unadjusted estimates and, if applicable, confounder-adjusted estimates and their precision (eg, 95% confidence interval).  Make clear which confounders were adjusted for and why they were included. | **Page 09, lines 248 - 289** |
| Main results | # 16b | Report category boundaries when continuous variables were categorized. | **Page 09, lines 261 - 267** |
| Main results | # 16c | If relevant, consider translating estimates of relative risk into absolute risk for a meaningful time period. | **Page 09, lines 248 - 267** |
| Other analyses | # 17 | Report other analyses done—eg analyses of subgroups and interactions, and sensitivity analyses. | **Page 10, lines 283 - 289** |
| **Discussion** |  |  |  |
| Key results | # 18 | Summarize key results with reference to study objectives. | **Page 10, lines 290 - 296** |
| Limitations | # 19 | Discuss limitations of the study, taking into account sources of potential bias or imprecision.  Discuss both direction and magnitude of any potential bias. | **Page 12, lines 357 - 366** |
| Interpretation | # 20 | Give a cautious overall interpretation considering objectives, limitations, multiplicity of analyses, results from similar studies, and other relevant evidence. | **Page 10, lines 297 - 356** |
| Generalizability | # 21 | Discuss the generalizability (external validity) of the study results. | **Page 13, lines 377 - 382** |
| **Other Information** |  |  |  |
| Funding | # 22 | Give the source of funding and the role of the funders for the present study and, if applicable, for the original study on which the present article is based. | **Page 13, lines 383 - 386** |

**Notes:**

• The STROBE checklist was distributed under the terms of the creative commons attribution license (CC-BY). This checklist was completed using 27. January 2023, using https://www.goodreports.org/, a tool developed by the EQUATOR Network in collaboration with Penelope.ai.
